# ZeroGEN: leveraging language models for zero-shot ligand design from protein sequences

**DOI:** 10.1093/bioinformatics/btaf572

**Published:** 2025-10-15

**Authors:** Yangyang Chen, Zixu Wang, Pengyong Li, Li Zeng, Xiangxiang Zeng, Lei Xu

**Affiliations:** College of Computer Science and Electronic Engineering, Hunan University, Changsha, Hunan 410082, P.R. China; College of Computer Science and Electronic Engineering, Hunan University, Changsha, Hunan 410082, P.R. China; School of Computer Science and Technology, Xidian University, Xian 710071, P.R. China; Department of AIDD, Shanghai Yuyao Biotechnology Co., Ltd., Shanghai 201109, P.R. China; College of Computer Science and Electronic Engineering, Hunan University, Changsha, Hunan 410082, P.R. China; School of Electronic and Communication Engineering, Shenzhen Polytechnic University, Shenzhen 518055, P.R. China

## Abstract

**Motivation:**

Deep generative methods based on language models have the capability to generate new data that resemble a given distribution and have begun to gain traction in ligand design. However, existing models face significant challenges when it comes to generating ligands for unseen targets, a scenario known as zero-shot learning. The ability to effectively generate ligands for novel targets is crucial for accelerating drug discovery and expanding the applicability of ligand design. Therefore, there is a pressing need to develop robust deep generative frameworks that can operate efficiently in zero-shot scenarios.

**Results:**

In this study, we introduce ZeroGEN, a novel zero-shot deep generative framework based on protein sequences. ZeroGEN analyzes extensive data on protein–ligand inter-relationships and incorporates contrastive learning to align known protein-ligand features, thereby enhancing the model’s understanding of potential interactions between proteins and ligands. Additionally, ZeroGEN employs self-distillation to filter the initially generated data, retaining only the ligands deemed reliable by the model. It also implements data augmentation techniques to aid the model in identifying ligands that match unseen targets. Experimental results demonstrate that ZeroGEN successfully generates ligands for unseen targets with strong affinity and desirable drug-like properties. Furthermore, visualizations of molecular docking and attention matrices reveal that ZeroGEN can autonomously focus on key residues of proteins, underscoring its capability to understand and generate effective ligands for novel targets.

**Availability and implementation:**

The source code and data of this work is freely available in the https://github.com/viko-3/ZeroGEN.

## 1 Introduction

Deep generative models have significantly expedited the drug discovery process (Zhavoronkov *et al.* 2019). Given a specific target protein, they aim to generate suitable ligands that effectively bind to a specific target protein. Traditional ligand generative models is typically conducted through two primary methods: (i) ligand-based drug design (Bacilieri and Moro 2006, Jin *et al.* 2018, Brown *et al.* 2019) generates novel molecules by studying known ligands distributions, which do not consider target information, resulting that the trained models only serve one target (Surade and Blundell 2012). Moreover, when faced with a target that has no ligand library, ligand-based drug design cannot be modeled. (ii) pocket-based methods generate novel molecules from 3D structural data of the protein pocket. The limitation is its dependence on high-resolution pocket structures of target proteins (Liang *et al.* 1998), restricting the use of targets without well-defined structures.

Given the above problems, protein sequence-based generative language models have emerged as a compromise. They are not entirely abandoning protein information like ligand-based approaches, nor do they overly rely on 3D pocket structural information, as pocket-based methods do. One major characteristic of protein sequence-based methods is that it generates molecules based solely on the sequence information of proteins. For instance, an early job (Grechishnikova 2021) treats the molecule generation task as a target-to-molecule machine translation task, generating molecules in a sequence-to-sequence manner [for ease of comparison at a later stage, we refer to it as Prot2Drug (Grechishnikova 2021) in this article]. DeepTarget (Chen *et al.* 2023) innovatively adapted conditional generative adversarial networks (cGANs) from the field of computer vision to the domain of molecular sequence generation. It leverages the strategic interplay between the generator and discriminator components to direct the synthesis of molecules that align with the specified target.

Previous research has demonstrated the feasibility of generating potential candidate ligands for protein sequences. However, as an increasing number of targetable proteins are being discovered, there is still a primary challenge that confronts current methods. The challenge is to generalize the model to a new protein sequence. These new proteins are not present in existing datasets and may lack known ligands for the model to learn from (Long *et al.* 2022). This scenario is typically referred to as zero-shot learning (Wang *et al.* 2019, Long *et al.* 2022). In this scenario, current methods often struggle to produce reliable ligands. By analyzing why existing protein sequence-based generative models are unsatisfactory in zero-shot scenarios, we found that these models do not construct a comprehension of the feature interaction relationship between the target sequence and the ligand. For instance, Prot2Drug simply translates protein sequence features into ligand sequences. DeepTarget is not a good training for the discriminator, although it uses a game approach to discriminate the positive or negative ligands. Not only that, the primary challenge of the zero-shot scenario stems from the requirement for the model to comprehend categories it has never encountered during training. These limitations present a significant challenge in generating reliable ligands through zero-shot scenarios.

To address the identified issues, we have structured our study around two principal questions: (i) How can the potential interaction relationship between proteins and ligands be determined? (ii) How can models be made adaptable to new protein data? In addressing the first question, we propose the integration of a contrastive learning mechanism alongside a cross-attention mechanism. Contrastive learning (He *et al.* 2020, Tian *et al.* 2020) aims to narrow the gap between feature representations of positive pairs while widening it for negative pairs. The cross-attention (Wei *et al.* 2020, Chen *et al.* 2021), a mechanism in deep learning for processing disparate types of data, enables the model to transfer and integrate information across different data modalities. We posit that generative language models employing both contrastive learning and cross-attention mechanisms can significantly enhance task performance by achieving a more profound comprehension of the feature relationship of data and improving model interpretability. To tackle the second question, we advocate for the adoption of a self-distillation-based data augmentation technique which bolsters the generation ability of models to novel categories. This technique involves producing pseudo labels on augmented samples from pre-trained models and refine the model using augmented samples. It can facilitate the learning process of the model and improve its ability to generalize.

**Figure 1.**
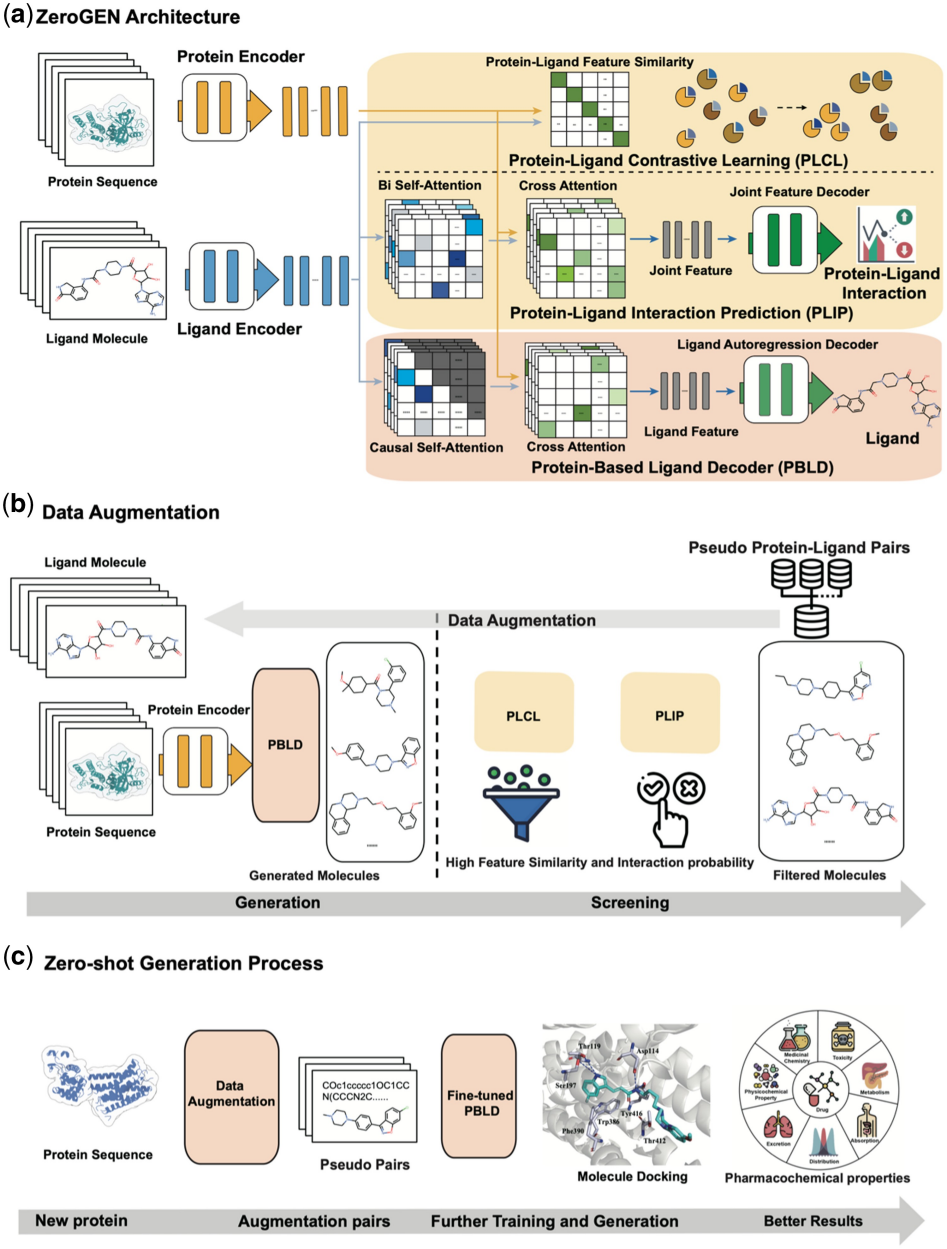
(a) The architecture of ZeroGEN. It consists of two parts: (i) the protein–ligand relationship extraction part contain a protein–ligand contrastive learning (PLCL) module and a protein–ligand interaction prediction (PLIP) module; and (ii) the ligand design part: protein-based ligand decoder (PBLD) module. The PLCL incorporates contrastive learning strategies for feature comparison of protein and ligand by similarity matrix. The PLIP employ a cross-attention mechanism to intricately extract the interactions between the fine-grained features of amino acids and molecule, further enhancing the ability of model to discern protein–ligand relationships. The PBLD generate ligands for giving protein sequence. (b) The data augmentation process is based on self-distillation. The ZeroGEN model can leverage the PLCL and PLIP to assess whether the generated molecules are relevant to the target protein by feature similarity and interaction probability. It can then filter out parts it deems irrelevant. The retained segments can further be used to augment a “pseudo” dataset toward the target protein. (c) The full generation process for ZeroGEN. A new protein sequence is encoded and decoded to several candidate ligands. By data augmentation, the new data pairs are used to re-train the model and get ligands with better affinity. (Icons used in this figure were obtained from Icons8 (https://icons8.com/) and are used under the Icons8 Free License).

Briefly, in this study, we introduce a deep generative language model aimed at zero-shot application named ZeroGEN. ZeroGEN presents four significant advancements: (i) ZeroGEN is specifically designed to tackle the challenges of ligand design in zero-shot scenarios, especially in settings where no structural or ligand information is available. It operates solely on amino acid sequences, making it applicable to novel or poorly characterized proteins, such as early-stage targets, or underexplored protein families with no known structures or ligand annotations. In these contexts, traditional docking or pocket-based methods are inapplicable or severely limited. By contrast, ZeroGEN can generate candidate ligands *de novo*, providing a complementary strategy that bridges the gap between sequence-only data and downstream structure-based refinement. Applying this method to four targets, we observed that ligands generated by ZeroGEN exhibit a higher affinity compared to those by previous methods. (ii) ZeroGEN analyzes a large amount of data on protein–ligand inter-relationships, integrating additional components for protein–ligand relationship extraction (Fig. 1a), which aid the model in capturing feature relationships through contrastive learning and a cross-attention layer. (iii) ZeroGEN proposes a data augmentation mechanism (Fig. 1b) based on self-distillation from protein–ligand relationship extraction. Relationship extraction components can assess the relevance of generated molecules to the target protein and filter out irrelevant ones. The remaining molecules serve as a “pseudo” dataset for the target, which is used to further fine-tune the generation module. This augmentation process aims to help the model understand which ligands match unseen targets and refine the model, and enhance generated ligands affinity toward the target protein, as illustrated in Fig. 1c. (iv) Docking simulations on the generated ligands confirmed the ability to interact with key residues of the target, which is a vital criterion for ligand design. Additionally, visualizing the attention matrix during the ligand generation process revealed the focus on the binding site between the target and the ligand during docking, thereby not only demonstrating the effectiveness of the model but also improving its interpretability in the context of deep learning.

## 2 Materials and methods

### 2.1 Model architecture

Inspired by above methods, here we introduce ZeroGEN, a comprehensive framework designed to learn possible relationships derived from protein–ligand pairs. To pre-train a unified model possessing both comprehension and generation capabilities, we propose a multi-modal mixture of encoder–decoder. The modules of the mix encoder–decoder are as follows:

#### 2.1.1 Protein encoder (PE) and ligand encoder (LE) 

These encoder follows the same structure as BERT(Vaswani *et al.* 2017, Acheampong *et al.* 2021), where a [CLS] token is place at the beginning of the text input, thereby providing an overall representation of the protein sequence or ligand sequence. An input protein sequence P is encoded into a sequence of embeddings: $\left\{p_{\mathrm{cls}},p_{1},\ldots ,p_{N}\right\}$. Input ligand L’s tokens $\left\{w_{1},\ldots ,w_{N}\right\}$ are encoded into a sequence of embeddings: $\left\{l_{\mathrm{cls}},l_{1},\ldots ,l_{N}\right\}$.

#### 2.1.2 Protein-based ligand feature extractor

The encoder incorporates protein data by incorporating an additional cross-attention (CA) (Wei *et al.* 2020) layer sandwiched between the self-attention (SA) (Zhao *et al.* 2020) layer and the feedforward network (FFN) within each transformation block of the molecular encoder. A task-specific [encoded] tag is added to the ligand sequence, and the [encoded] output embedding is harnessed as a multi-modal representation of the protein–ligand pair. An input ligand sequence is encoded into a sequence of embeddings: $\left\{l_{p\_\mathrm{cls}},l_{p\_1},\ldots ,l_{p\_N}\right\}$.

#### 2.1.3 Protein-grounded ligand decoder

The decoder substitutes the bidirectional self-attention layer in the protein feature-based text encoder with a causal self-attention layer. A [decoded] token signifies the start of a ligand sequence, while an end-of-sequence token is used to denote its termination.

As shown in Fig. 1a, during the pre-training phase, we collectively optimized three objectives, consisting of two comprehension-based objectives and one generation-based objective. To be specific, two encoder modules based on transformer layers are employed to encode the sequences of proteins and ligands separately. The global features of proteins and ligands are then fed into the protein–ligand contrastive learning (PLCL) module for feature comparison. Positive protein–ligand pairs and negative pairs that are challenging to differentiate are selected in the contrastive learning phase to be input into the protein–ligand interaction prediction (PLIP) module. The PLIP module applies a cross-attention mechanism to intricately extract interactions, thereby further enhancing the ability to discern protein–ligand relationships. Simultaneously, a protein-based ligand decoder (PBLD) is designed to learn the generation of ligands capable of interacting with proteins, utilizing known protein–ligand sample pairs and a cross-attention mechanism. The training process consists of three loss functions, as follows:

#### 2.1.4 Protein-ligand contrastive learning (PLCL) loss

The protein–ligand contrastive learning (PLCL) module is a central component of ZeroGEN. It enables the model to learn a shared latent space in which representations of known protein–ligand pairs are pulled closer, while mismatched pairs are pushed apart. This alignment is essential for capturing the underlying interaction semantics between protein sequences and ligand structures, especially in the absence of explicit structural information. During pre-training, PLCL helps the model build a biologically meaningful embedding space that generalizes to unseen targets in zero-shot settings. Additionally, PLCL plays a critical role in the self-distillation process by providing a similarity-based scoring function for filtering generated molecules. Specifically, it estimates the feature similarity between generated ligands and target proteins, allowing the model to select high-confidence pseudo-labeled data. This dual usage of PLCL highlights its importance not only in learning protein–ligand relationships, but also in guiding reliable data augmentation and improving generalization.

This loss aims to align the latent feature space of the protein transformer and the ligand transformer by encouraging positive protein–ligand pairs to have similar representations in contrast to the negative pairs (Radford *et al.* 2021). It has been shown to be an effective objective for improving multi-modal task. This loss learns a similarity function $s=g_{p}{\left(p_{\mathrm{cls}}\right)}^{T}g_{l}(l_{\mathrm{cls}})$, where $g_{p}$ and $g_{l}$ are linear transformations that map embeddings to normalized representations. Two queues are maintained to store the last M protein–ligand pair representations from the momentum encoders. The normalized features from the momentum encoders are represented as $g_{p}^{q}(p_{\mathrm{cls}}^{q})$ and $g_{l}^{q}(l_{\mathrm{cls}}^{q})$. The similarity function can be redefined as $s(P,L)=g_{p}{\left(p_{\mathrm{cls}}\right)}^{T}g_{l}^{q}(l_{\mathrm{cls}}^{q})$ and $s(L,P)=g_{l}{\left(l_{\mathrm{cls}}\right)}^{T}g_{p}^{q}(p_{\mathrm{cls}}^{q})$.

For each protein and ligand, the softmax-normalized protein-to-ligand and ligand-to-protein similarity score is calculated as:


$$f_{m}^{p2l}(P)=\frac{\exp\left(s(P,L_{m})/\tau \right)}{\sum_{b=1}^{M}\exp\left(s(P,L_{b})/\tau \right)},$$

$$f_{m}^{l2p}(L)=\frac{\exp\left(s(L,P_{m})/\tau \right)}{\sum_{b=1}^{M}\exp\left(s(L,P_{b})/\tau \right)},$$

where τ is a learnable parameter. The PLCL loss is defined as the cross entropy H between the ground-truth one-hot similarity ($y^{p2l}(P)$ and $y^{l2p}(L)$):


$$L_{\text{PLC}}=\frac{1}{2}E_{(P,L)∼D}[H(y^{p2l}(P),f^{p2l}(P))+H(y^{l2p}(L),f^{l2p}(L))].$$

#### 2.1.5 Protein–ligand interaction prediction (PLIP) loss

Protein–ligand interaction prediction loss (PLIP) for binary classification. This loss function is grounded in the activation of a protein-grounded molecule encoder, which aims to learn a protein–ligand multi-modal representation that captures the binary classification of protein–ligand binding. The PLIP for binary classification views the problem as a classification task where the PLIP head (a logistic layer) predicts the likelihood of protein–ligand pair interaction. In this context, the loss function learns a binary classification function $f^{b}(P,L)=g_{b}(l_{p\_\mathrm{cls}})$, where $g_{b}$ is linear transformation. The prediction is a binary label representing the presence or absence of an interaction. For each protein and ligand, the binary interaction prediction can be calculated as:


$$L_{\mathrm{PLM}}^{\text{binary}\;}=-E_{(P,L)∼D}[y^{b}(P,L)\log(f^{b}(P,L))+(1-y^{b}(P,L))l\mathrm{og}(1-f^{b}(P,L))]$$

where $y^{b}(P,L)$ is the binary ground-truth label indicating whether the interaction is present (1) or absent 0 in the dataset.

#### 2.1.6 Protein-based ligand decoder (PBLD) loss

This function is the key component of our task, generating relevant molecules for a given protein sequence. It optimizes a cross-entropy loss, training the model to maximize the likelihood of the molecule sequence in an autoregressive manner. The generation loss can be formulated as:


$$L_{\text{GM}}=-E_{(P,L)∼D}\sum_{i=1}^{i=N}\log p(w_{i}|w_{<i},p_{1},\ldots ,p_{N})$$

### 2.2 Data augmentation from self-distillation

The process is illustrated in Fig. 1b. Initially, we take a protein sequence as input and input it into the PBLD. The PBLD generates a set of relevant molecule sequences based on the characteristics of the input protein. Next, we combine the generated valid molecules with the corresponding source protein targets. These pairs are then fed into the PLCL. The PLCL leverages this input to calculate the feature similarity scores between the protein and each molecule, indicating their feature potential distance:


$$s=g_{p}{\left(p_{\mathrm{cls}}\right)}^{T}g_{l}(l_{\mathrm{cls}})$$

where $g_{p}$ and $g_{l}$ are linear transformations that map embeddings to normalized representations. To guarantee the meaningfulness of the generated pseudo-data pairs, we pre-calculated the feature similarity distributions for each target by separately analyzing 10 000 randomly selected inactive molecules and known active molecules. This preliminary step ensures the ability of PLC to differentiate between inactive and active molecular. We established the 95% locus of the distribution of inactive molecules as the screening threshold. Subsequently, protein–molecule pairs surpassing this threshold were retained to form a new dataset. For more detailed screening, we can further use PBPL to predict the interaction of dataset and remain the high interaction probably pairs.

Overall, this approach enables the augmentation of data through the self-distillation process, allowing for more robust training and addressing the challenges associated with limited data availability.

### 2.3 Evaluation metrics

To assess the performance of ZeroGEN in ligand generation for unseen targets, we employed several evaluation metrics:

**Docking score**: We used AutoDock Vina to compute the docking score of each generated ligand with its respective protein. The docking score (measured in kcal/mol) estimates the binding free energy, with lower (more negative) values indicating stronger predicted binding affinities. This score is widely used as a surrogate for binding strength in virtual screening.
**Tanimoto similarity**: To evaluate chemical similarity, we computed the Tanimoto similarity between generated ligands and known active compounds using Extended-Connectivity Fingerprints (ECFPs). A Tanimoto coefficient above 0.5 is commonly considered indicative of structural resemblance.
**Uniqueness and novelty**: Uniqueness is defined as the proportion of non-duplicate molecules among the generated set, and novelty as the proportion of molecules not found in the training dataset or ChEMBL database.
**Protein–ligand feature similarity**: In the self-distillation process, we calculated the feature similarity between protein and ligand embeddings using the PLCL module. A higher similarity score suggests stronger relevance between the generated ligand and the target protein. We used this metric to select high-quality pseudo pairs for data augmentation.

These metrics together evaluate both the bioactivity and chemical quality of generated ligands, as well as their novelty and interpretability.

## 3 Results

### 3.1 ZeroGEN framework

The proposed ZeroGEN framework is shown in Fig. 1a. The multiple modules work together to make ligand design in the zero-shot scenario more promising. ZeroGEN is equipped with a protein–ligand relationship understanding part. This part is based on both coarse-grained contrastive learning and fine-grained interaction analysis for modeling protein–ligand relationships. Specifically, we employ two encoder modules based on transformer layers to encode the sequences of proteins and ligands separately. Then, the global features of proteins and ligands are fed into the protein–ligand contrastive learning (PLCL) module for feature comparison. Subsequently, we select positive protein–ligand pairs and negative pairs that are challenging to differentiate in the contrastive learning phase to input into the protein–ligand interaction prediction (PLIP) module. PLIP applies cross-attention mechanism to intricately extract the interactions, thereby further enhancing the ability to discern protein–ligand relationships. Simultaneously, PBLD is designed to learn the generation of ligands capable of interacting with proteins, utilizing known protein–ligand sample pairs and a cross-attention mechanism. The attention map from the cross-attention layer reveals how the model extracts feature from the protein sequences that are most relevant to the generation process, thereby lending a degree of interpretability to the generated results. The parameters across ZeroGEN modules largely overlap, allowing the feature knowledge learned through different tasks to be transferred and integrated.

Furthermore, to further enhance its performance in zero-shot scenarios, we have designed an augmentation process that utilizes the protein–ligand relationship understanding part. Since it is equipped with both feature relationship extraction and generative parts, ZeroGEN has the capacity to refine its generated outcomes, which is helpful under the conditions of zero-shot learning. In Fig. 1b, the initially generated molecules and corresponding targets are screened, leaving molecules with more similar feature spaces as new data to further optimize the model. This process can be regarded as a form of self-distillation, leveraging solely the knowledge produced by the model itself, thereby eliminating the necessity for external model guidance. Summarily, in zero-shot scenarios, ZeroGEN employs a generative process plus augmentation based on self-distillation approach to adjust the sampling direction of the generator, as shown in Fig. 1c. After utilizing the augmented “pseudo ligands” to adjust the generative direction, ZeroGEN can generate ligands more specifically tailored to the target protein. For clarity in the subsequent article, we will refer to the version of the model that includes the data augmentation and fine-tuning operation as ZeroGEN and to the version without these enhancements as ZeroGEN-Vanilla.

### 3.2 ZeroGEN-Vanilla can generate molecules with better affinity for target

Before introducing the self-distillation process into the model, we first assess the effectiveness of the ZeroGEN-Vanilla model architecture. We compare ZeroGEN-Vanilla with three cases: Prot2Drug (a baseline treats the ligand design of target as a translation of a protein sequence into a drug sequence), DeepTarget (a baseline applies GANs to design ligands from protein sequence) and Randomization (randomly selected molecules, if the model does not work well in the ZERO-SHOT scenario, it can be viewed as random sampling). We select four targets [DRD2 (Wang *et al.* 2018), AKT1 (Wu *et al.* 2010), GSK3β (Arnost *et al.* 2010), ROCK1 (Green *et al.* 2015)] without relevant molecules (removed from the training data) and extract the sequence from the PDB structures (6cm4, 3o96, 3l1s, and 4yve) in the literature to generate molecules. These targets were chosen for their pharmacological relevance and structural diversity. DRD2 is a representative target, while AKT1, GSK3β, and ROCK1 are protein kinases involved in various signaling pathways and disease mechanisms. Additionally, these proteins have well-characterized crystal structures, and their ligands are documented in public databases such as ChEMBL. We removed all ligands associated with these targets from the training data to construct strict zero-shot evaluation scenarios, ensuring that the model had not previously seen ligand information for these proteins. As baselines for comparison, we generate the same valid molecules for Prot2Drug, DeepTarget, and Randomization.

**Figure 2.**
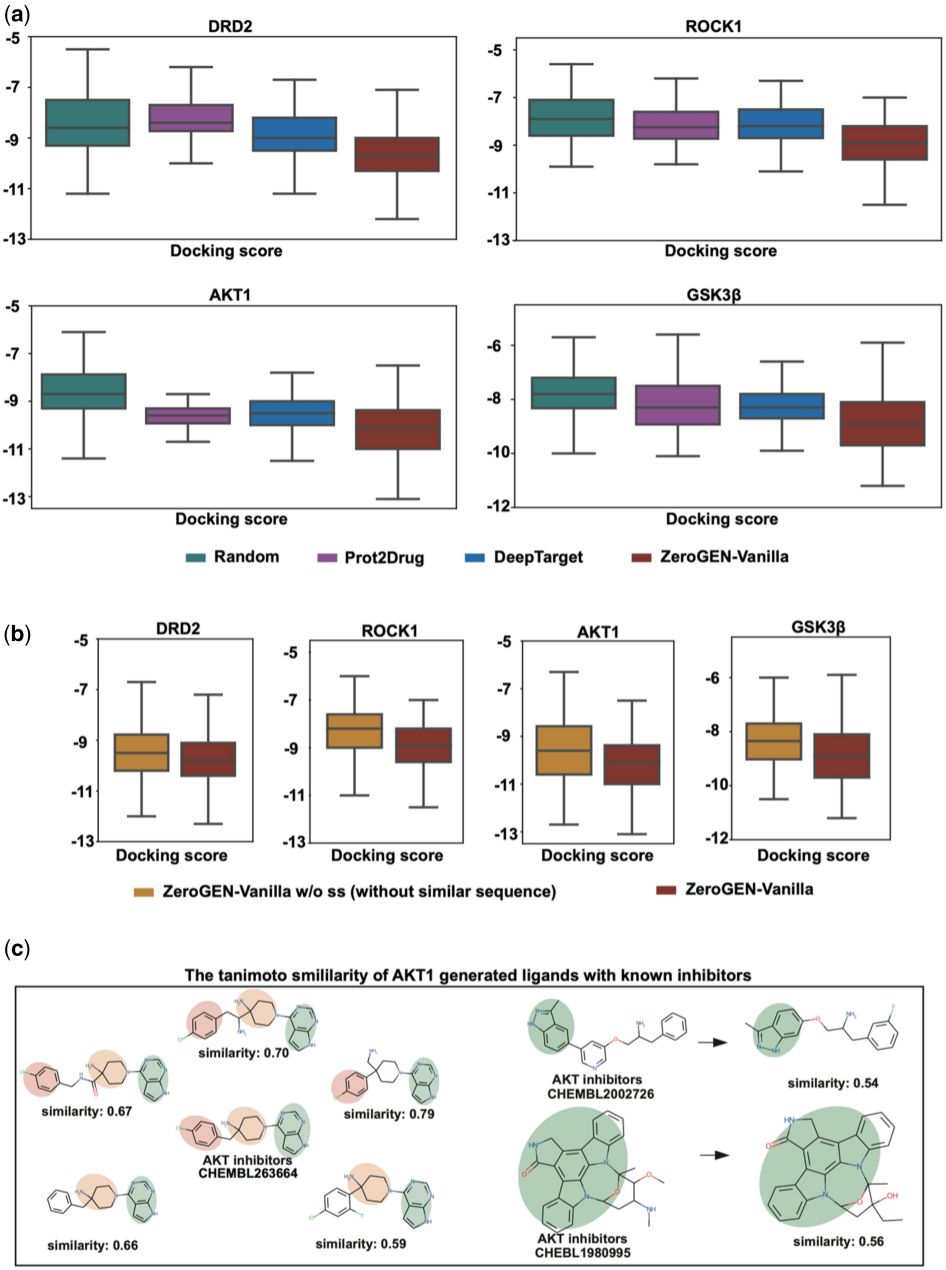
(a) The generate ligands docking scores of four targets (DRD2, ROCK1, AKT1, and GSK3β) for randomization, Prot2Drug, DeepTarget, and ZeroGEN-Vanilla. (b) The generate ligands docking scores of four targets for ZeroGEN-Vanilla w/o ss (without similar sequence) and ZeroGEN-Vanilla. (c) The high tanimoto similarity of molecules generated by ZeroGEN-Vanilla with known AKT1 inhibitors.

The binding affinity of generated ligands is important for the target-aimed models. To evaluate the ability, for each target, 100 valid and unique generated molecules are docked and docking scores are calculated by AutoDock Vina (Trott and Olson 2010, Alhossary *et al.* 2015). As shown in Fig. 2a, the randomly selected molecules clearly differentiate from other models, having the almost worst docking distribution. It means that randomly selected molecules do not serve as credible drug candidates. DeepTarget has a better mean score compared to randomization, while there is no cap increase. The performance of Prot2Drug is inconsistent, but still underperforms overall. In contrast to the former, ZeroGEN-Vanilla presents considerable results compared to the former. The distribution has shown a notable improvement, with not only the best scores increasing but also the overall average scores experiencing a significant upward shift, reflecting positively on the effectiveness of ZeroGEN-Vanilla.

Further, as outlined in the data construction in Section 2, we utilize a clustering algorithm to identify similar protein sequences. To demonstrate the effectiveness of the cluster method, we also evaluate the model that removed the same cluster protein sequence for each target (a total of 2239 data pairs); the same cluster ID with target proteins is also removed to train a new model called ZeroGEN-Vanilla w/o ss. In Fig. 1, available as supplementary data at *Bioinformatics* online, we use biopython (Cock *et al.* 2009) to assess the maximum similarity between the target sequence and others within the training set across two scenarios. The analysis reveals that eliminating the similar protein sequence results in each target exhibiting a lower similarity than when only the target itself is removed. For the results of ZeroGEN-Vanilla w/o ss in Fig. 2b, we can observe that erasing similar protein sequences had a negative effect, generating molecules with worse docking scores. In this regard, it is reasonable to believe that as a sequence-based model, ZeroGEN-Vanilla autonomously learns information about relevant molecules from similar sequences within the dataset, thereby being utilized by the target protein.

Next, we analyze the above valid and unique generated molecules for similarity with known active compounds. The high similarity of ECFP fingerprints to known active compounds suggests that model can generate similar substructures or topological features. In this part, we employ some known AKT inhibitors as case studies. The left of Fig. 2c shows an Akt inhibitor for enhancing chimeric t cell persistence(Patent ID: WO-2021102038-A1, CHEMBL263664) (McHardy *et al.* 2010). It has been observed that can generate five molecules with high tanimoto similarity and same scaffolds to known inhibitors, which are the target sequence not yet included in the training set (eight molecules ZeroGEN-Vanilla generated have high similar scaffolds with this inhibitor, tanimoto similarity more than 0.5). Likewise, we also apply molecules generated from ZeroGEN-Vanilla w/o ss to match some known inhibitors. Although ZeroGEN-Vanilla w/o ss did not trained on sequence data highly similar to AKT1, it still successfully generates few molecules with similar scaffolds to known inhibitors (CHEMBL2002726 and CHEMBL1980995) (the right of Fig. 2c).

### 3.3 Data augmentation based on self-distillation can further improve the models

**Figure 3.**
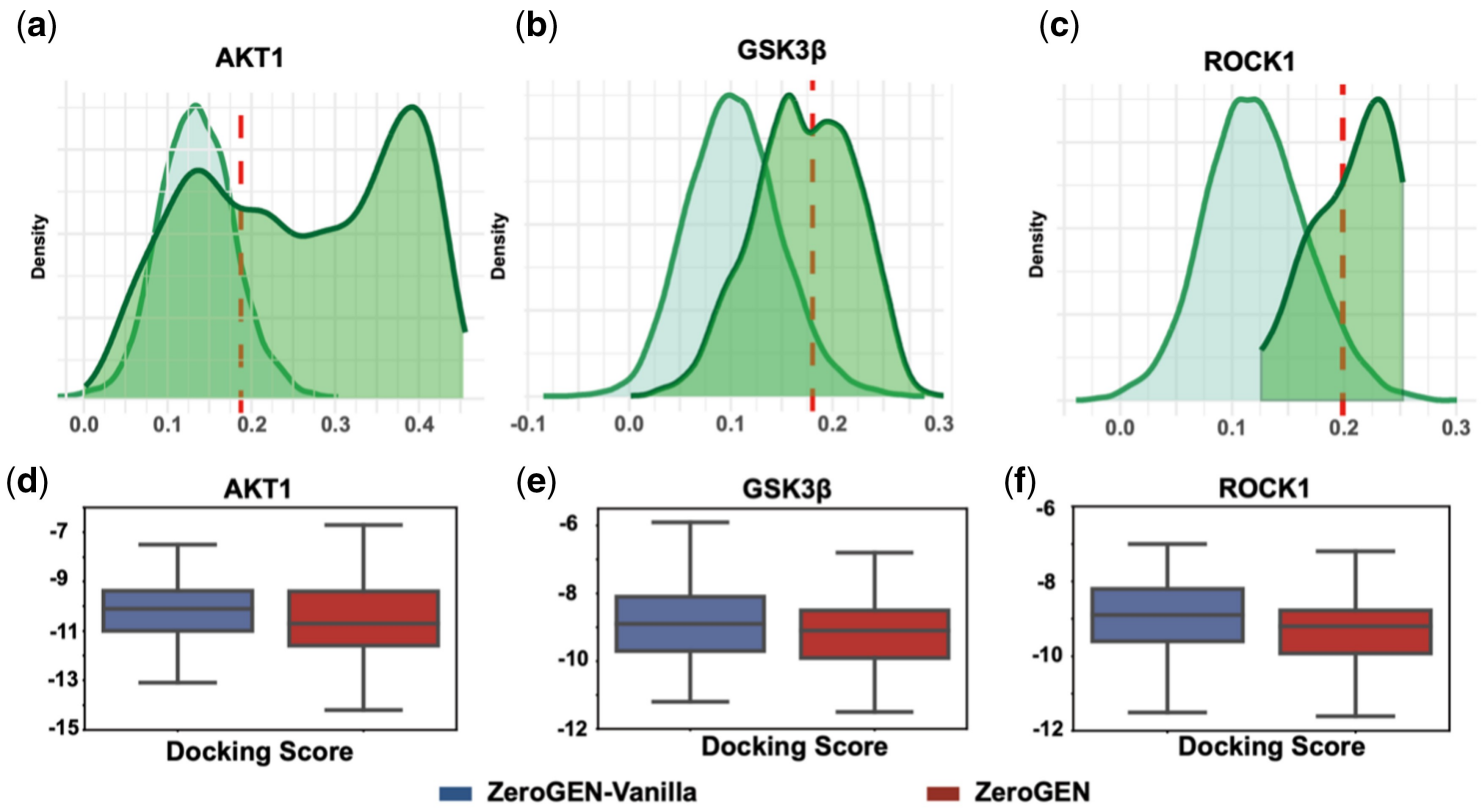
(a–c) Distribution of ligand and protein feature similarity calculated by the PLCL module from ZeroGEN-Vanilla. The two distributions represent randomly selected inactive molecules and known active molecules, respectively. The dashed vertical line indicates the 95th percentile of the similarity distribution of inactive molecules to the target sequence. (d–f) The docking scores of ligands generated by ZeroGEN-Vanilla and ZeroGEN.

Data augmentation enhances the performance of the model in the presence of data constraints, and this method can expand the training set and improve the training efficiency of the model without relying on additional external data. Unlike traditional data augmentation, under zero-shot, there are no reference samples. Thus, we propose an augmentation process based on self- distillation (Zhang *et al.* 2019, Li *et al.* 2022). An effective self-distillation method should have the ability to distinguish between active and inactive molecules of the target. To evaluate whether ZeroGEN-Vanilla can distinguish relevant from irrelevant ligands, we selected known active molecules for each target and randomly sampled 10 000 inactive compounds from the ChEMBL database. Using the PLCL module, we computed the protein–ligand feature similarity for all molecules. Since no active ligands are available in a true zero-shot scenario, we constructed a background similarity distribution using the inactive molecules and defined the 95th percentile as a confidence threshold. This target-specific threshold enables us to identify and retain only high-confidence generated ligands that are unlikely to belong to the inactive space, thereby facilitating more reliable pseudo-labeling for self-distillation. As described in Section 1, the PLCL and PLIP modules can help the model to extract the feature relationship between ligand and protein sequence. In a preliminary discrimination test between active and inactive molecules, we revealed that the feature similarity distributions derived from the PLCL module exhibit greater discrimination between active and reactive molecules. Figure 3a–c depicts the feature similarity distributions from PLCL of molecules with the target sequence. The active molecules can obviously be different from inactive molecules, with a higher similarity distribution. For example, the active molecules of ROCK1 have feature similarities exceeding 0.12, while inactive molecules are distributed around 0.1. Based on this discriminating ability, we propose a data augmentation method to target. Specifically, the data augmentation is shown in Fig. 1b. The molecules generated by ZeroGEN-Vanilla are calculated the feature similarity with target by the PLCL and screened. Due to the absence of available data in the zero-shot scenario, it is impossible to ascertain the distribution characteristics of active molecules. Consequently, for every target, the 95th percentile of the similarity distribution among inactive molecular features is established as the screening criterion. From the generated data, we select 1000 molecules per target that exceed this threshold as augmented data. This fixed number is chosen to balance confidence and diversity, ensuring that the self-distillation process does not propagate low-quality or overly similar ligands. While we do not claim biological activity for these molecules, their elevated feature similarity scores serve as model-internal confidence proxies in the absence of labeled data. The filtered molecules will be used to fine-tune the ZeroGEN-Vanilla model, resulting in the refined ZeroGEN model. The results of molecular docking in Fig. 3d–f indicate that the docking scores generated by ZeroGEN can be further enhanced by ZeroGEN-Vanilla through the data augmentation process. In addition, we continue to investigate the impact of the data augmentation process when removing similar protein sequences. Figure 2a–c, available as supplementary data at *Bioinformatics* online implies that even though similar protein sequences were removed, PLCL was still able to distinguish between active and inactive molecules to some extent. Although not as good as ZeroGEN-Vanilla, in Fig. 2d–f, available as supplementary data at *Bioinformatics* online, the data augmentation process can also improve docking affinity.

In addition to evaluating the quality of the generated molecules through molecule docking, we also measure the relationship between the generated molecules and known active molecules from the perspective of drug discovery. Here, we adopt two methods to measure the relationship between the generated ligands and known ligands: (i) the number of generated molecules with a similarity greater than 0.5 to known active molecules in ChEMBL (GenMol >0.5 Sim), and (ii) the number of known active small molecules identified using a similarity 0.5 threshold (ActMol Identified with > 0.5 Sim). Each target is evaluated with 10 000 valid generated molecules. In Table 1, the generated molecules have high novelty and are unique, avoiding evaluating a large number of identical generated samples. Then, we can observe that ZeroGEN not only improve the number of generated molecules with high tanimoto similarity (the distributions of tanimoto similarity are shown in Fig. 3, available as supplementary data at *Bioinformatics* online), but also expand the model generation space. Likewise, the same processes also act on ZeroGEN-Vanilla that removes similar protein sequences. In Table 1, available as supplementary data at *Bioinformatics* online, it also has better performance than before. A point worth explaining is that the number of known ligands corresponding to ROCK1 is only 19, so only a few of the ligands in Table 1 can correspond to (the known ligands corresponding to the three targets are presented in Table 2, available as supplementary data at *Bioinformatics* online).

**Table 1.** The number of generated molecules with a similarity >0.5 to known active molecules in ChEMBL (GenMol >0.5 Sim) and the number of known active small molecules identified using a similarity 0.5 threshold (ActMol identified with > 0.5 Sim).

Target	ZeroGEN-Vanilla				ZeroGEN			
	Unique	Novelty	GenMol >0.5 Sim	ActMol identified with > 0.5 Sim	Unique	Novelty	GenMol >0.5 Sim	ActMol identified with > 0.5 Sim
AKT1	1.0	0.9996	188	295	1.0	0.9999	613	331
GSK3B	1.0	1.0	301	228	1.0	0.9999	663	301
ROCK1	1.0	1.0	1	1	1.0	1.0	1	2

### 3.4 ZeroGEN can generate molecules recognizing key residues of targets

**Figure 4.**
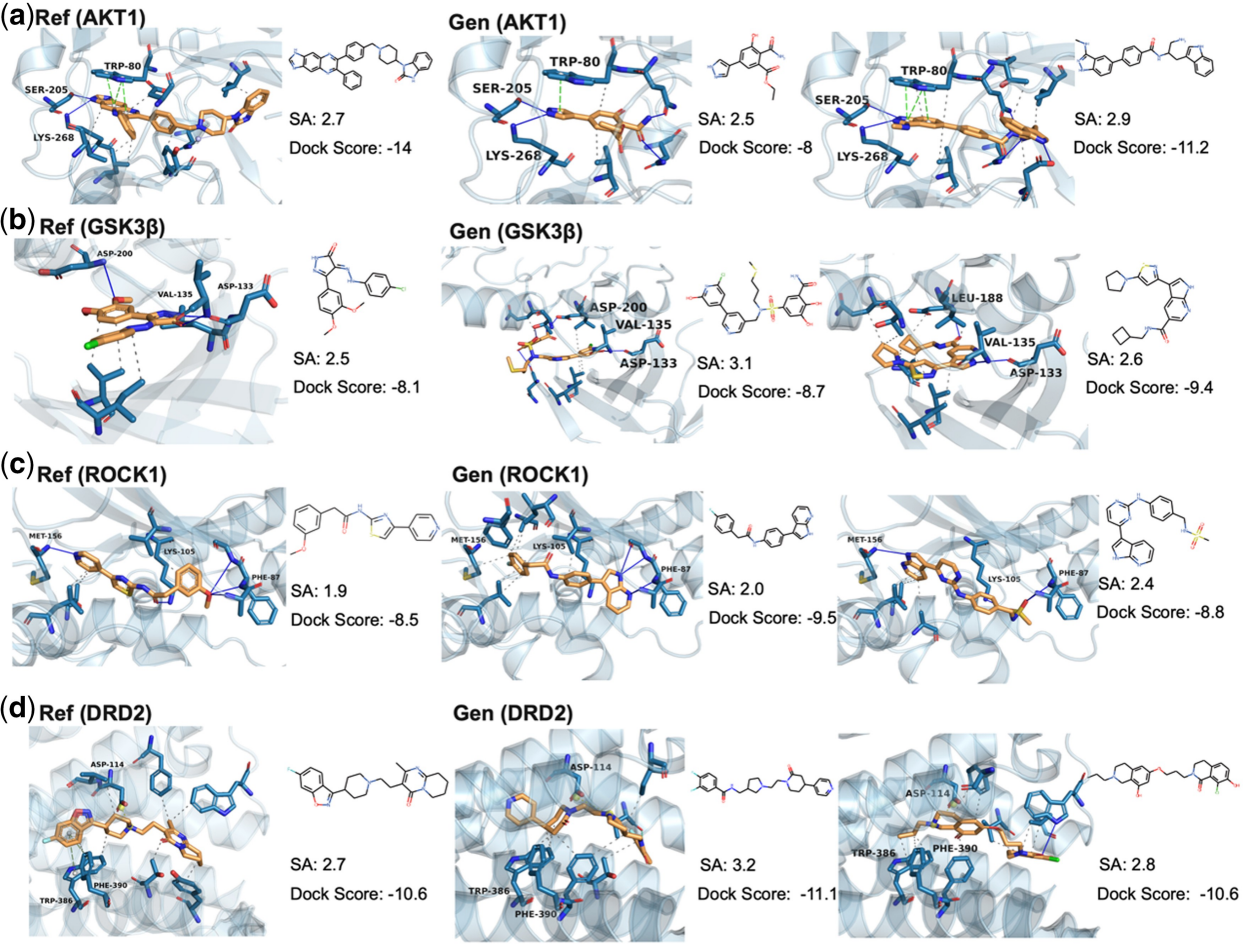
Docking cases selected with reference active compounds and the key residues from the literature. (a) Cases for AKT1. In the protein–ligand complexes belonging to the active compound and the generating molecule, respectively, the ligand forms hydrogen bonds with SER-205 and LYS-268 and engages in π–π stacking with TRP-80. (b) Cases for GSK3β. The ligand establishes hydrogen bond interactions with the side chains of ASP-200, VAL-135, and ASP-133. (c) Cases for ROCK1. The ligand engaged in hydrogen bonding with residues MET-156, LYS-105, and PHE-85 of the protein. (d) Cases for DRD2. ASP-114 forms a salt bridge and TRP-386, PHE-390 engage in hydrophobic interactions with the ligand.

In this section, we firstly further analyze the docking of the generated molecules to the target. We have extracted key residues for target-molecule interactions in crystal structures from the relevant literature. Similar binding mode can demonstrate the ability of our model to reproduce active compounds. In the docked complex illustrated in Fig. 4a, the active compound establishes hydrogen bonds with Serine 205 (SER-205) and Lysine 268 (LYS-268) (Wu *et al.* 2010), which serve to stabilize its position within the active site. Moreover, an aromatic π–π stacking interaction between the ligand and Tryptophan 80 (TRP-80) enhances the binding specificity and affinity, indicating a robust ligand–protein interaction network essential for inhibitory activity. To broaden the scope of our evaluation, we included three additional protein targets—VEGFR2, TGFB1, and BRD4—not present in the original experiments. These targets represent diverse structural and functional categories and were selected from a recent benchmark study on generative models(Zhu *et al.* 2023). The results, available in Figs 6 and 7, available as supplementary data at *Bioinformatics* online, further support the model’s ability to generalize across unseen protein classes. These interactions are also observed for the generated molecules. Likewise, generated molecules for other target also share interactions with the same amino acid residues as the reference ligands, which indicates that the model-generated molecules are capable of fitting into the binding sites of the reference ligands.

Given the promising performance of ZeroGEN, we further investigated whether the model can identify key residues involved in ligand binding. Since ZeroGEN leverages cross-attention in the PBLD, we interpreted the attention weights from the generated ligand tokens to each amino acid as indicators of residue importance during generation. By aggregating attention weights across all ligand tokens and attention heads, we obtained a single attention score for each residue. These scores were then mapped to the 3D structure of the target protein using PyMol. Residues with top 15% attention scores were highlighted in red (Fig. 4, available as supplementary data at *Bioinformatics* online), revealing that the model consistently focuses on regions near known binding pockets. Notably, ZeroGEN was trained using only protein sequences without any structural or pocket annotations. Since binding pockets often result from long-range interactions and are not localized in sequence space, this observation suggests that the model has learned to infer structurally meaningful features from sequence alone, providing a degree of interpretability.

These findings underscore the advanced capability of our model in deciphering the structural and functional intricacies of proteins. By leveraging the attention mechanism, ZeroGEN not only demonstrates an adeptness in learning from complex biological datasets but also unveils residues that are crucial for the binding affinity and biological activity within the active site of protein. This is particularly significant in the realms of drug design and protein engineering, where accurate identification of these pivotal residues directly influences the development of novel therapeutics and the modification of protein functions.

### 3.5 ZeroGEN can generate molecules satisfying ADMET properties

The success of a drug candidate hinges critically on its high potency and selectivity, along with a favorable absorption, distribution, metabolism, excretion, and toxicity (ADMET) profile. In this study, we selected 1000 molecules generated by our model to evaluate their drug-like properties, based on the standards established by ADMET Lab 2.0 (Xiong *et al.* 2021). As illustrated in Fig. 5, available as supplementary data at *Bioinformatics* online, the analysis encompasses key pharmacokinetic properties and toxicity constraints for drug candidates, revealing that the generated molecules align with these benchmarks. This suggests that the molecules produced by our model meet the fundamental criteria required for drug candidates.

## 4 Conclusion

In this study, we propose ZeroGEN, a ligand design model in zero-shot scenarios from protein sequences. ZeroGEN neither relies on the structural features of the target nor directly applies redundant sequence features. Through contrastive learning and cross-attention mechanisms, it can obtain fine-grained features and further optimize the feature space. By incorporating a self-distillation process, ZeroGEN provides a data augmentation method to enhance its zero-shot learning capabilities, improving the affinity of the ligand. Importantly, unlike structure-based generative frameworks that depend on known binding pockets or co-crystal structures, ZeroGEN can be applied in early discovery stages where such information is lacking. This makes it particularly suitable for first-pass ligand design in unexplored targets. Through docking simulations and visualization of the attention matrix, we further demonstrate that ZeroGEN is able to capture critical information about ligand-target binding, providing interpretability.

In the absence of structural or pocket-level annotations, ZeroGEN employs a modular architecture in which each component is tailored to a specific subtask within the zero-shot ligand generation pipeline. The contrastive module aligns global protein and ligand embeddings to capture coarse-grained interaction patterns, while PLIP and PBLD apply residue-level attention to model local interactions. Each component addresses a distinct subtask, and shared transformer encoders help reduce computational cost. Future work may improve biological specificity by incorporating pocket-aware masking, residue-weighted contrastive learning, or structure-informed pre-training, and enhance efficiency through model distillation or modular inference.

## Supplementary Material

btaf572_Supplementary_Data

## Data Availability

All data and implementation details of code can be obtained from github (https://github.com/viko-3/ZeroGEN).
