# Supplementary material for: ZeroGEN: leveraging language models for zero-shot ligand design from protein sequences
Source: Bioinformatics. 2025 Oct 15;41(11):btaf572. doi: 10.1093/bioinformatics/btaf572 (PMC12596140; doi:10.1093/bioinformatics/btaf572)
Supplement: btaf572_Supplementary_Data [file btaf572_supplementary_data.docx]

# Supplementary information

## Dataset Construction

The training ligand–protein pairs were collated from the ChEMBL database[26]. Proteins are composed of amino acid sequences and ligands are represented by Simplified Molecular-Input Line-Entry System (SMILES). After conducting following preliminary screening:

1. The median value of all affinity scores of the molecules corresponding to each target was taken and those greater than the median value were considered active ligands.
2. Invalid SMILES molecular formulas were removed using RDKit[29], including removing salts and stereochemistry from the SMILES sequence. Inorganic molecules or those that could not be interpreted by RDKit were removed.
3. Proteins with unknown token “X” were removed.
4. Characters of SMILES containing the isomer information were removed, and the strings containing the following tokens were reserved.

$$\{C,c,N,n,S,s,O,o,F,\mathrm{Cl},[nH],\mathrm{Br},1,2,3,4,5,6,7,8,9,\#,=,+,-,(,),[,]\}$$

the data comprises 535,476 active ligand–protein pairs. Each protein and each ligand form a data pair.

Because of our model is based on sequence information, in subsequent experiments, we want to verify whether similar protein sequences would have a positive effect on the model. There is an assumption that analogous inhibitors can act on proteins with resemblance, and such proteins share similar sequence representations. We posit that the presence of other protein sequences in the training set, which exhibit high similarity to the target protein sequence, contributed positively to the training. Thus, a cluster method is applied to the protein sequences in our study. Same as the MONN[30], we use the single-linkage clustering algorithm[31]. The cluster distance between a pair of proteins $\left( P_{i},P_{j} \right)$ is defined as:

$$Distance\left( P_{i},P_{j} \right)=1-\frac{SW \left( P_{i},P_{j} \right)}{\sqrt{\left( SW\left( P_{i},P_{i} \right)SW\left( P_{j},P_{j} \right) \right)}}$$

where $SW \left( P_{i},P_{j} \right)$ stands for the Smith-Waterman alignment score calculated based on the SSW library[32]. Then, we use hierarchical clustering to assign clusters to each protein sequence, with 0.6 as the threshold. In our experiments, we will exclude sequences that are either similar to the target protein sequence or to the target protein itself (in same cluster), as applicable, to ascertain the influence of similar sequences on the performance.

## Experiments setting.

To evaluate the zero-shot learning ability of ZeroGEN, we removed the relevant protein-ligand pairs for the tested proteins. The removed data were treated as the test set, while the remaining data constituted the training set. The training set has 539,425 protein-ligand pairs and test set has 9,936 protein-ligand pairs. The initial training epoch was set to 50, with a learning rate of 0.0001. For self-distillation, we fine-tuned the model for 10 epochs. During training, PLIP and PBLD shared all parameters except for the causal self-attention layers. During inferring and sampling, all parameters are frozen.

## Supplementary Figures and Tables


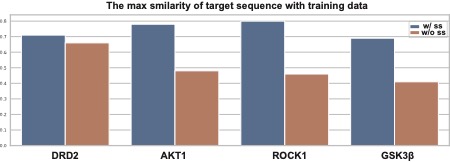


**Supplementary Figure 1** The four targets correspond to the maximum similarity of protein sequences under ZeroGEN-Vanilla and ZeroGEN and other protein sequences in the training set.


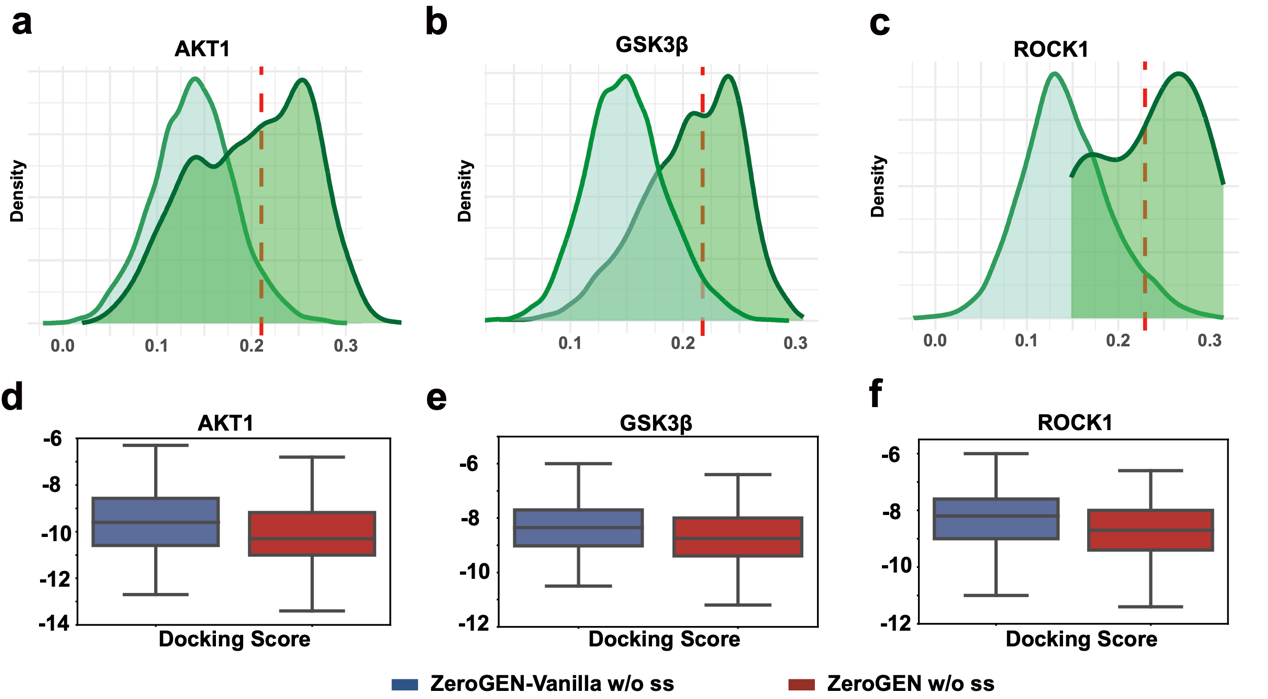


**Supplementary Figure 2** **a-c.** Distribution of ligand and protein feature similarity calculated by the PLCL module from ZeroGEN-Vanilla w/o ss. Light green is randomly selected inactive molecules, dark green is known active molecules. The red dashed line represents the 95th percentile of the similarity distribution of randomly selected inactive molecules to the target sequence. **d-f.** The docking scores of ligands generated by ZeroGEN-Vanilla w/o ss, and ZeroGEN w/o ss.


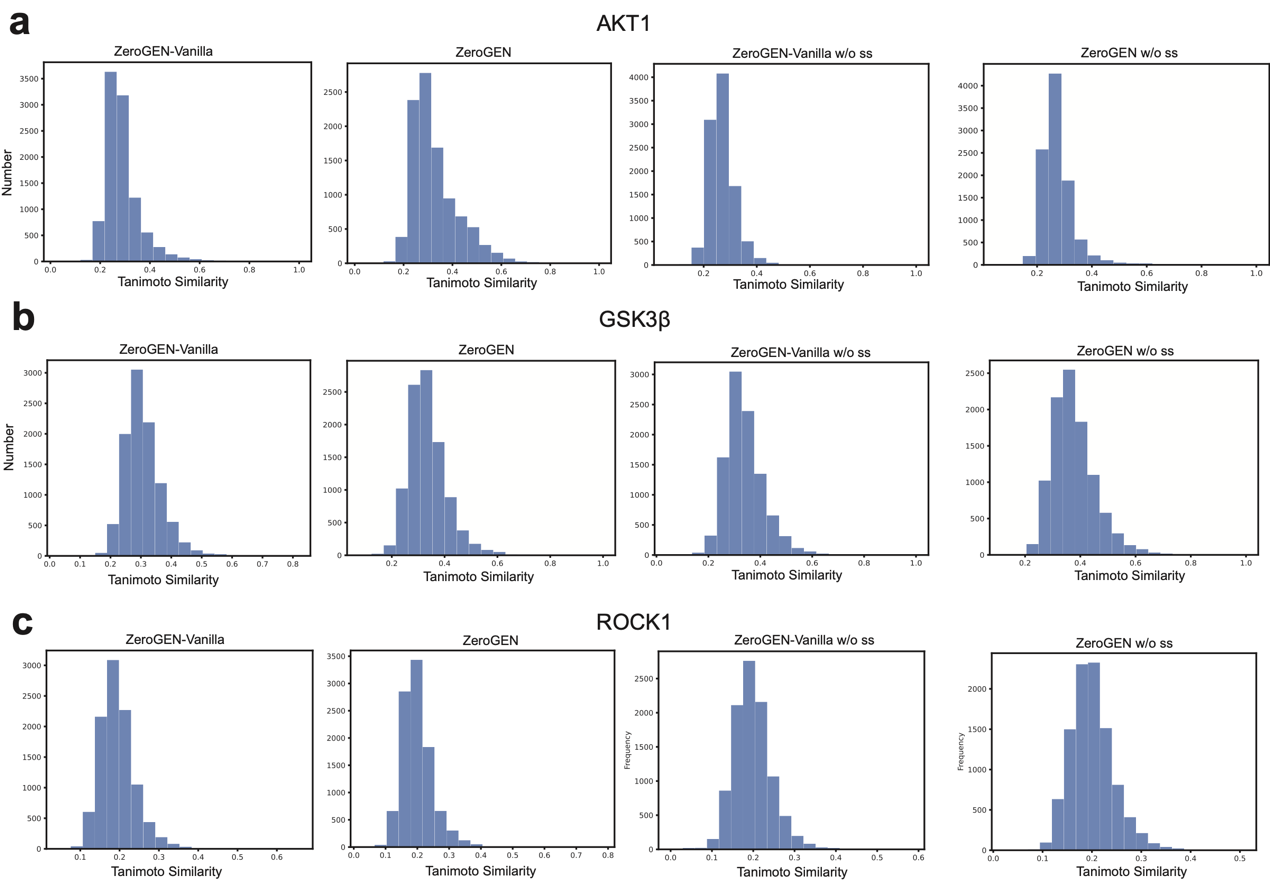


**Supplementary Figure 3** **a.** Maximum tanimoto similarity distribution of generated molecules to known active ligands for AKT1. **b.** Maximum tanimoto similarity distribution of generated molecules to known active ligands for GSK3β. c Maximum tanimoto similarity distribution of generated molecules to known active ligands for ROCK1.


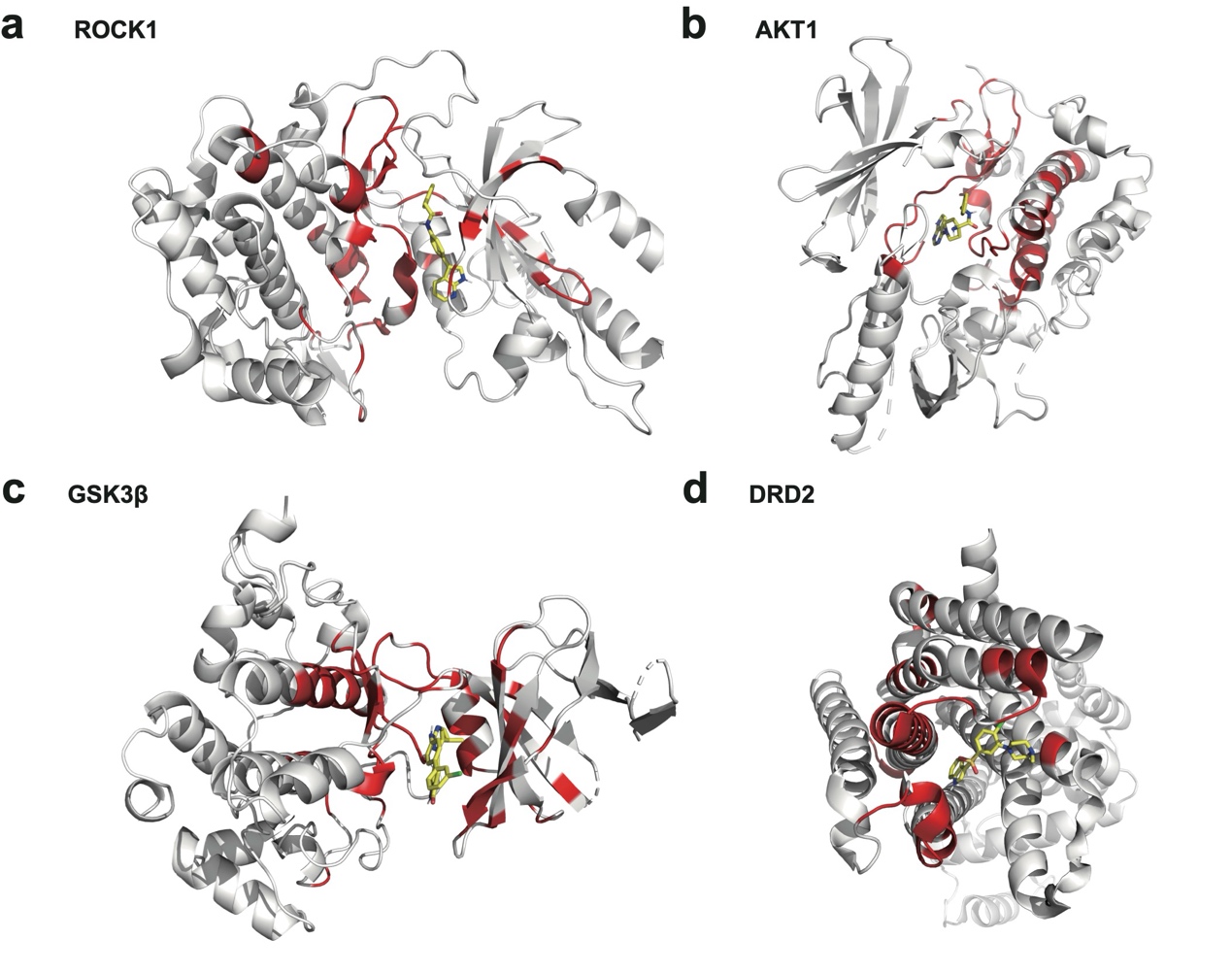


**Supplementary Figure 4** Visualization of the weights of the attention layer in PBLD when the target generates the corresponding molecules. Residues with the weight of more than 85% are highlighted in red, denoting areas of high significance as determined by the attention weights of the model.


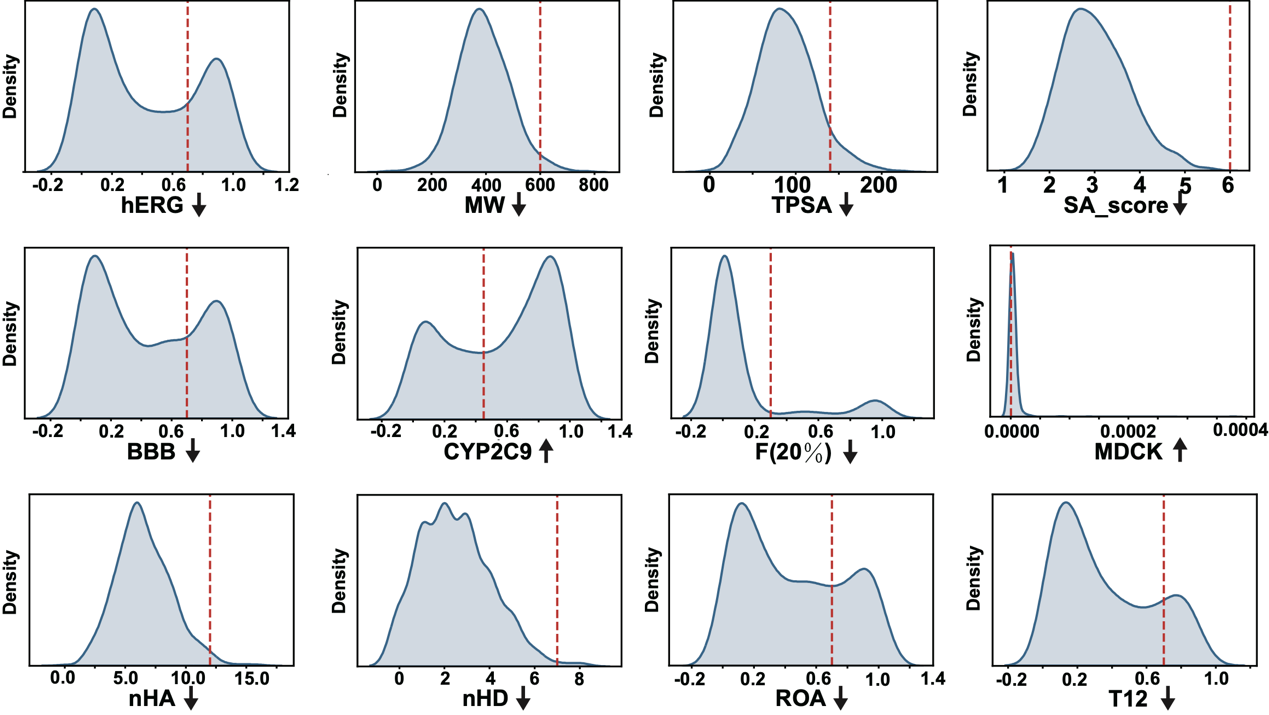


**Supplementary Figure 5** Distributions of the ADMET properties of the generated by ZeroGEN. The dashed lines indicate the property thresholds, where an upward arrow signifies a preference for values exceeding the threshold, and a downward arrow denotes a preference for values below the threshold. TPSA, the topological polar surface area, is optimal within the range of 0–140 (Å^2^); MW, indicating molecular weight, is ideal between 100–600; nHA, the number of hydrogen bond acceptors, should range from 0–12; nHD, the number of hydrogen bond donors, is preferred to be 0–7; SA the synthetic accessibility score, is considered suitable if less than 6; the predicted Madin–Darby Canine Kidney cells (MDCK) measures the uptake efficiency of a drug into the body, suitable when: >2 × 10^−6^ (cm/s); BBB, indicating the likelihood of a drug crossing the blood-brain barrier, has a qualified range of 0–0.7; F(20%), predicting the probability of human oral bioavailability being less than 20%, is considered acceptable if under 0.3; CYP2C9 evaluates drug metabolism, specifically the probability of inhibiting reactions; T12 assesses the drug's half-life, with the desired probability being for a half-life of ≤3 hours; hERG tests for cardiac toxicity, specifically the probability of inhibiting the human ether-a-go-go gene; ROA evaluates acute mammalian toxicity, with the value indicating the probability of toxicity.


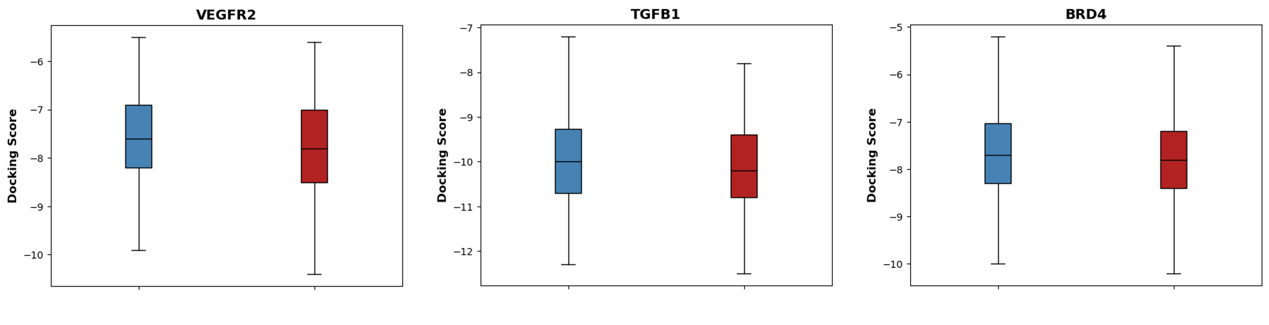


**Supplementary Figure 6** The generate ligands docking scores of three targets for ZeroGEN-Vanilla w/o ss (without similar sequence) and ZeroGEN-Vanilla.

**Supplementary Figure 7** Docking cases selected with reference active compounds and the key residues from the literature. **a.** Cases for VEGFR2. In the protein-ligand complexes belonging to the active compound and the generating molecule, respectively, the ligand forms hydrogen bonds with ASP-1044 and CYS-917 **b.** Cases for TGFB1. The ligand establishes hydrogen bond interactions with the side chains of H15-283. **c.** Cases for BRD4. The ligand engaged in hydrogen bonding with residues ASN-140 of the protein.

**Supplementary Table 1** The number of generated molecules with a similarity greater than 0.5 to known active molecules in chembel (GenMol >0.5 Sim) and the number of known active small molecules identified using a similarity 0.5 threshold (ActMol Identified with>0.5 Sim).

|  | ZeroGEN-Vanilla w/o ss | | | | ZeroGEN w/o ss | | | |
| --- | --- | --- | --- | --- | --- | --- | --- | --- |
|  | Unique | Novelty | GenMol >0.5 Sim | ActMol Identified with>0.5 Sim | Unique | Novelty | GenMol >0.5 Sim | ActMol Identified with>0.5 Sim |
| AKT1 | 1.0 | 0.9999 | 35 | 49 | 1.0 | 1.0 | 115 | 110 |
| GSK3B | 1.0 | 0.9996 | 98 | 130 | 1.0 | 0.9997 | 286 | 228 |
| ROCK1 | 1.0 | 1.0 | 2 | 1 | 1.0 | 1.0 | 3 | 2 |

**Supplementary Table 2** The number of known ligands for protein.

| Protein | The number of known ligands |
| --- | --- |
| AKT1 | 2089 |
| GSK3B | 3877 |
| ROCK1 | 19 |
